# Multi-Scale Fusion Lightweight Target Detection Method for Coal and Gangue Based on EMBS-YOLOv8s

**DOI:** 10.3390/s25061734

**Published:** 2025-03-11

**Authors:** Lin Gao, Pengwei Yu, Hongjuan Dong, Wenjie Wang

**Affiliations:** 1 Mechanical Engineering School, Inner Mongolia University of Science and Technology, Baotou 014010, China; dongao521922@163.com (L.G.); 13020433750@163.com (P.Y.); wwj@mail.nwpu.edu.cn (W.W.); 2 Mining and Coal School, Inner Mongolia University of Science and Technology, Baotou 014010, China

**Keywords:** YOLOv8s, coal gangue detection, CLAHE, efficient multi-branch and scale feature pyramid network (EMBSFPN), Wise-SIoU loss function

## Abstract

The accurate detection of coal gangue is an important prerequisite for the intelligent sorting of coal gangue. Aiming at existing coal gangue detection methods, which have problems such as low detection accuracy and complex model structure, a multi-scale fusion lightweight coal gangue target detection method based on the EMBS-YOLOv8s model is proposed. Firstly, the coal gangue images collected through the visual dark box platform are preprocessed using CLAHE to improve the contrast and clarity of the images. Secondly, the PAN-FAN structure is replaced by the EMBSFPN structure in the neck network. This structure can fully utilize the features of different scales, improve the model’s detection accuracy, and reduce its complexity. Finally, the CIoU loss function is replaced by the Wise-SIoU loss function at the prediction end. This improves the model’s convergence and stability and solves the problem of the imbalance of hard and easy samples in the dataset. The experimental results show that the mean average precision of the EMBS-YOLOv8s model on the self-constructed coal gangue dataset reaches 96.0%, which is 2.1% higher than that of the original YOLOv8s model. The Params, FLOPs, and Size of the model are also reduced by 29.59%, 12.68%, and 28.44%, respectively, relative to those of the original YOLOv8s model. Meanwhile, the detection speed of the EMBS-YOLOv8s model is 93.28 f.s^−1^, which has certain real-time detection performance. Compared with other YOLO series models, the EMBS-YOLOv8s model can effectively avoid the occurrence of false detection and missed detection phenomena in complex scenes such as low illumination, high noise, and motion blur.

## 1. Introduction

As one of the important energy sources in the global energy consumption structure, coal resources are essential in the domains of the iron and steel, electric power, transportation, and chemical industries [1,2]. However, the coal mining or sorting process often contains part of the gangue, which not only reduces the efficiency of coal combustion but also means that the gangue will emit harmful gasses after burning, polluting land and water sources and so on [3,4,5]. Therefore, to improve the development and utilization efficiency of coal resources and reduce the harm caused by gangue, the accurate detection and sorting of coal gangue is of great significance.

Traditional coal gangue sorting methods currently include manual, mechanical, and ray sorting. Among them, the manual sorting method is greatly influenced by subjective human factors and is prone to leakage and misselection. In addition, the manual sorting site environment is harsh on workers’ physical and mental health, causing severe impact. The mechanical separation method includes the heavy medium method and the jigging method. Although this method has high accuracy and saves manpower, it consumes a lot of water resources, and the sewage generated after separation also causes serious pollution in the environment. The x-rays and γ-rays in the ray sorting method are high-energy radiation sources, which cause severe damage to the health of workers in the event of leakage [6,7].

With the development of artificial intelligence technology in recent years, it has also been widely used in coal gangue sorting. It mainly includes two coal gangue detection methods based on traditional machine learning and deep learning. The coal gangue detection method based on traditional machine learning manually extracts the features of coal gangue images. Then, it inputs these features into a support vector machine [8,9], BP neural network [10], random forest [11], and other classification methods for training to achieve the purpose of classification and recognition. This method can solve the problems of the harm to human health, high cost, low accuracy, and low resource utilization of traditional coal gangue sorting methods to a certain extent. However, with the continuous improvement of the coal industry’s requirements on detection accuracy and real-time performance, such techniques have gradually exposed their limitations, such as poor adaptability to complex working conditions and reliance on manual experience for feature extraction [12,13,14].

In contrast, the coal gangue detection method based on deep learning has significant advantages. The method automatically learns complex features from the coal gangue image through the multi-layer convolutional layer and pooling layer, which reduces the subjectivity and dependency of manually extracted features. It is precisely because of this characteristic that the coal gangue detection method based on deep learning has stronger generalization ability and higher detection accuracy [15,16,17]. Pengfei Shan et al. [18] performed a dark channel dehazing operation on coal gangue images, which enabled the improved CBAM Faster R-CNN model to achieve a detection accuracy of 90.44%. However, the accuracy rate depended on the effect of the preprocessed coal gangue image. Yasong Gao et al. [19] replaced the SE module in the MobileNetV3 large model with the CBAM module, which improved the detection accuracy of the improved model. However, the CBAM module had more parameters, which led to a relative increase in the number of parameters in the improved model. Lei Zhang et al. [20] proposed a GCEB-YOLO model based on the YOLOv5 model by introducing the GhostNet network, the BiFPN structure, and so on. The improved model drastically reduced the number of parameters and computation of the original model. However, the accuracy was slightly lower, at 91.4%. Qinghua Mao et al. [21] improved the detection speed of the model based on the YOLOv7 model by replacing the ordinary convolution in the backbone network with depth-separable convolution. However, the extensive use of depth-separable convolution resulted in a decrease in detection accuracy of 92.8%. Fangfang Xin et al. [22] effectively improved the detection accuracy of the model by introducing the SE module, 3D expansion, and the DCN module on the basis of YOLOv8n. However, the application of the above modules leads to a large number of parameters in the improved model and slower speed.

In summary, the existing coal gangue detection methods make it difficult to achieve a balance between detection accuracy and lightweight models. Most of the algorithms are for the pursuit of high accuracy, and the model design is particularly complex, resulting in large algorithm parameter counts occupying a large amount of memory. Therefore, to address the above problems, this paper proposes a multi-scale-fusion lightweight coal gangue detection method based on the EMBS-YOLOv8s model. The following are this paper’s primary contributions:

(1) Aiming at the problems of the low contrast and clarity of coal gangue images caused by low illumination, high conveyor belt speed, and dust interference in the coal separation environment, this paper uses Contrast-Limited Adaptive Histogram Equalization (CLAHE) to preprocess the acquired images of coal gangue to improve the quality of the images.
(2) The self-designed Efficient Multi-Branch and Scale Feature Pyramid Network (EMBSFPN) is used in the neck network of the YOLOv8s model, which improves the detection accuracy of the model and also reduces the complexity of the model.
(3) Replacing the CIoU loss function with the Wise-SIoU function at the prediction end of the YOLOv8s model improves the convergence and stability of the model and solves the problem that there is an imbalance of hard and easy samples in the dataset.

The rest of this paper is organized as follows: Section 1 describes the modules to which the improved model is applied. Section 2 describes the coal gangue image acquisition and preprocessing operations. Section 3 describes the experimental environment and the evaluation indicators. Section 4 analyzes the experimental results. Finally, Section 5 summarizes this paper.

## 2. EMBS-YOLOv8s Model

The YOLOv8s model is improved based on the YOLOv5s model, so the algorithm structure is similar to that of the YOLOv5s model, which is divided into four modules: the input, backbone, neck, and head [23]. However, compared with the YOLOv5s model, the YOLOv8s model replaces the C3 module in the backbone network of the original YOLOv5s model with the C2f module, which enhances the model feature extraction capability while reducing computation and the number of parameters. Another major improvement is replacing the traditional Anchor-based detection head with a decoupled head structure without anchors, which makes the model more adaptable and flexible to changes in the size and shape of the target, and the accuracy and convergence speed of the model is better. In addition, Ultralytics, the company that developed the YOLOv8s model, proposed a new YOLOv11s model in 2024. This model builds on the YOLOv8s model by introducing new C3k2 and C2PSA modules in the backbone network [24]. Although the YOLOv11s model’s feature extraction capability is improved, the introduction of new modules makes the model structure complex, resulting in the YOLOv11s model not performing as well as YOLOv8s in real-time coal gangue target detection tasks. In summary, considering that a good model needs to be high-accuracy, lightweight, and real-time and have other advantages, we chose the YOLOv8s model as the basic model to be improved in this paper.

**Figure 1:**
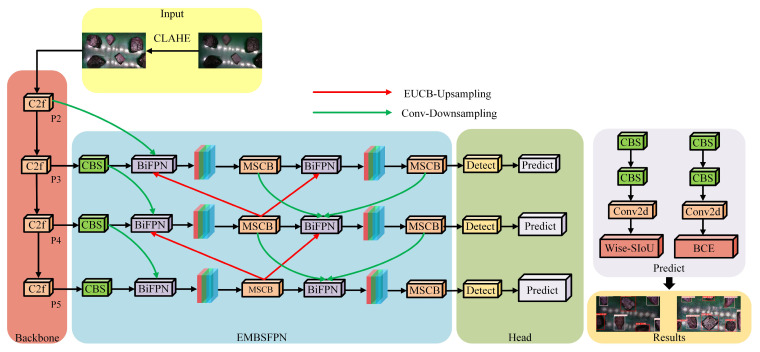
EMBS-YOLOv8s model structure.

The structure of the EMBS-YOLOv8s model is shown in Figure 1 below. The specific improvement measures are as follows: Firstly, the PAN-FPN structure in the neck network of the original YOLOv8s model is replaced by the EMBSFPN structure, which improves the model’s detection accuracy and reduces the model’s complexity. Secondly, the CIoU loss function in the prediction end is replaced by the Wise-SIoU function, which improves the convergence and stability of the model and solves the problem of an imbalance of hard and easy samples in the dataset.

### 2.1. Improvements to the Neck Network

**Figure 2:**
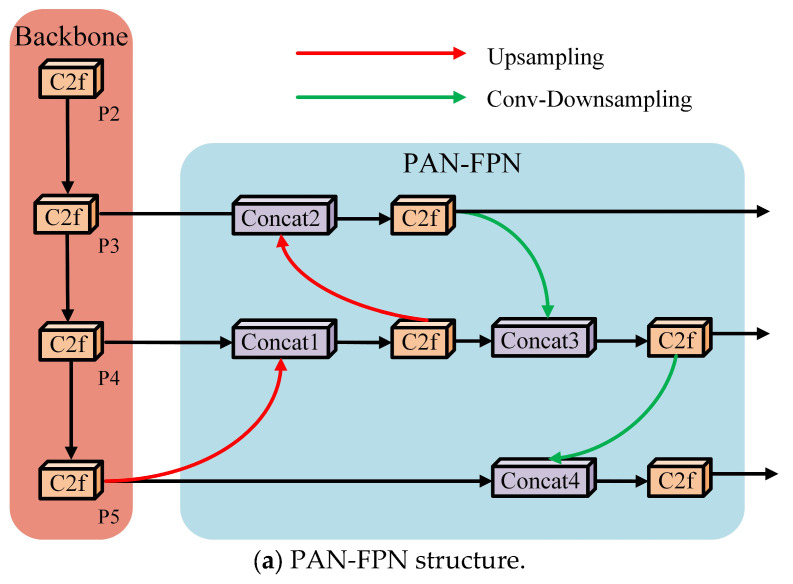

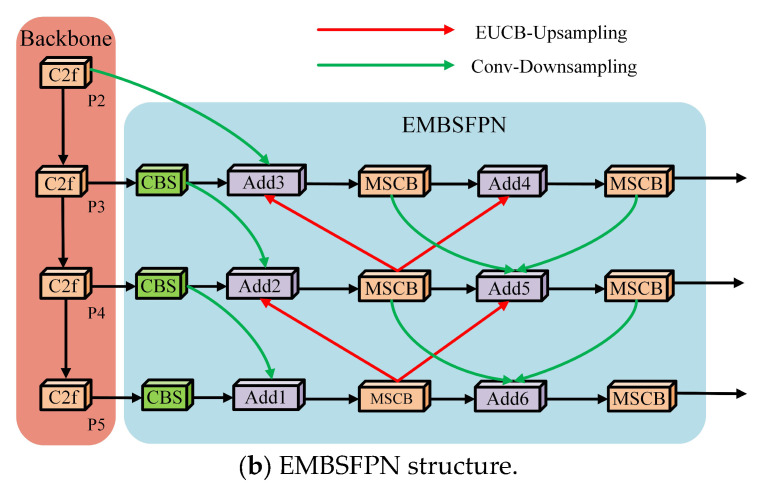
Diagrams of the PAN-FPN structure and BiFPN structure.

The neck network in the original YOLOv8s model uses a combination of PAN-FPN structures for feature fusion. The PAN-FPN structure is a significant improvement in the YOLO algorithm family made after the YOLOv4 version, which greatly improves the model’s accuracy. As can be seen from Figure 2a, the PAN-FPN structure fuses different levels of feature maps through two different paths, top–down and bottom–up, to form a pyramid that can generate multi-scale features. Specifically, in the top–down path, the PAN-FPN structure passes the semantic feature information from the deep layer to the shallow layer through upsampling. In the bottom–up path, the PAN-FPN structure passes the shallow localization feature information to the deep layer through downsampling. Finally, the feature maps of the two paths are fused to obtain the final multi-scale feature that contains both semantic and localization feature information. However, the fusion method of this structure tends to be the fusion between the same scale features and lacks the comprehensive processing and fusion of the multi-scale information of different resolution layers. And, the number of parameters of this structure is high. Therefore, in this paper, the PAN-FPN structure in the original model structure is replaced by the EMBSFPN structure.

The EMBSFPN structure is shown in Figure 2b. As can be seen from the figure, unlike the original PAN-FPN structure which tends to be fused between same-scale features, the EMBSFPN structure is a combination of deep feature information with shallow feature information at the same level as well as at high resolutions, which allows for the retention of rich feature information. For example, in Concat1 of the PAN-FPN structure, its input source consists of the sibling P4 layer and the P5 layer after upsampling. However, it ignores the importance of the shallow localization feature information in the P3 layer. In contrast, in Add2 of the EMBSFPN structure, its input source is a P3 layer after downsampling in addition to the sibling P4 layer and the P5 layer after upsampling. Thus, in Add2, there is both fusion between same-scale features and fusion between multi-scale features of different resolution layers. In deeper network structures, such as Add5, information from four different layers can be fused at the same time, which significantly improves the performance of medium-sized targets. The EMBSFPN structure makes full use of the multi-scale information from different resolution layers in the way described above, which effectively improves the detection accuracy of the model. In addition, the EMBSFPN structure replaces the original channel splicing method, Concat, with the Add method, which reduces the number of parameters and computation while also performing an adaptive selection of weighted fusion by the importance of features at different scales. In order to match this approach, this paper carries out the channel alignment operation before feature fusion; that is, the CBS operation in Figure 2b is utilized to reduce the number of channels.

The EMBSFPN structure also improves on the PAN-FPN structure by introducing new modules. These include the improvement of the C2f module into the multi-scale convolution block (MSCB) and the improvement of the upsampling method into the efficient up-convolution block (EUCB) [25]. They are described in detail below.

#### 2.1.1. Multi-Scale Convolution Block (MSCB)

**Figure 3:**
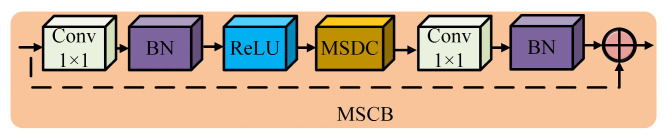
Schematic diagram of MSCB structure.

The structure of the MSCB is similar to the Inverted Residuals and Linear Bottlenecks (IRB) structure in MobileNetV2 [26]. However, compared with the IRB structure, the MSCB innovatively introduces a multi-scale depth-wise convolution (MSDC) structure. The MSDC structure enables the model to perform deep convolution operations at multiple scales at the same time to extract rich multi-scale features. In addition, the MSCB also uses channel shuffling operations [27] to enable the model to effectively integrate information at different scales, thus significantly enhancing the expression ability of feature maps. The specific structure of the MSCB is shown in Figure 3 below. As can be seen from the figure, we first use 1 × 1 point-by-point convolution to extend the channel dimension of the feature map as a way to enrich the feature expression capability. Second, we use batch normalization (BN) and ReLU activation function to accelerate the training process and enhance the nonlinear expression capability of the model. After that, we use MSDC to capture the information of the multi-scale features and perform fusion. Finally, another set of 1 × 1 point-by-point convolution and BN operations are used to convert back to the channel dimensions of the original feature maps to obtain the fused final features.

**Figure 4:**
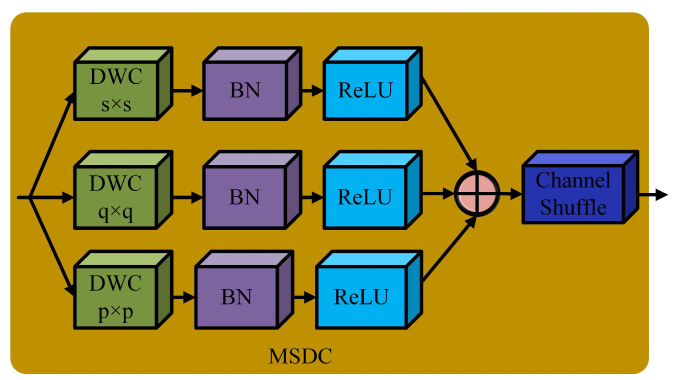
Schematic diagram of MSDC structure.

Referring to the heterogeneous kernel selection (HKS) mechanism in YOLO-MS [28], the MSDC structure introduces deep and large convolutional kernels of the sizes 5, 7, and 9. These convolution kernels of different sizes can adaptively match features of different scales and gradually obtain the information of multi-scale features in the processing process so as to improve the feature extraction ability of the model. The schematic diagram of the MSDC structure is shown in Figure 4. When a feature passes through the MSDC structure, the structure first determines the most suitable convolution kernel based on the scale of the feature and then proceeds with the subsequent operations. Next, it fuses the convolution results of these different scales. In this way, the final multi-scale feature, rich in information, is formed.

#### 2.1.2. Efficient Up-Convolution Block (EUCB)

**Figure 5:**
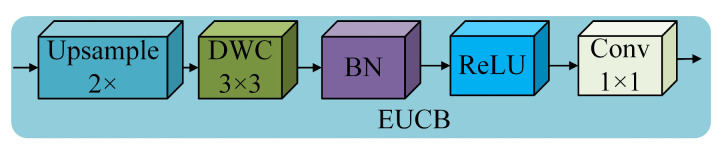
Schematic diagram of EUCB structure.

EUCB uses an efficient up-convolution block to upsample the feature maps of the current stage level by level. This process enables the enhancement of the fusion of feature information between different levels and stages, as it makes the dimension and resolution of the current-stage feature maps match those of the feature maps at the next connection. The EUCB structure is illustrated in Figure 5. As can be seen from Figure 5, the EUCB structure first enlarges the input feature map scale by a factor of 2 through the upsampling operation, which enables the model to capture more details of the features. Second, 3 × 3 depthwise convolution is applied to efficiently extract the features and enhance the expressive capability without significantly increasing the computational effort. After that, BN and ReLU activation function operations are performed to accelerate the speed of network convergence and increase the nonlinear expressive capability of the network. Finally, 1 × 1 convolution is performed to reduce the number of channels so that the feature maps after the upsampling are downsized to match the number of channels in the next stage.

### 2.2. Improvement of Loss Function

The original YOLOv8s model adopts the CIoU loss function at the prediction end. Compared with the traditional IoU loss function, the biggest advantage of the CIoU loss function is that while considering the overlapping area of the bounding box, it also considers the distance from the center point and the aspect ratio. CIoU loss function solves the problem that the traditional IoU loss function still provides the moving direction of the bounding box when the prediction box and the real box do not intersect. However, the CIoU loss function calculates all the loss variables as a whole and does not take into account the mismatch between the actual target frame and the prediction frame orientation, which leads to slow and unstable model convergence. In addition, there may be the problem of producing worse models due to the arbitrary matching of the prediction frames during training.

**Figure 6:**
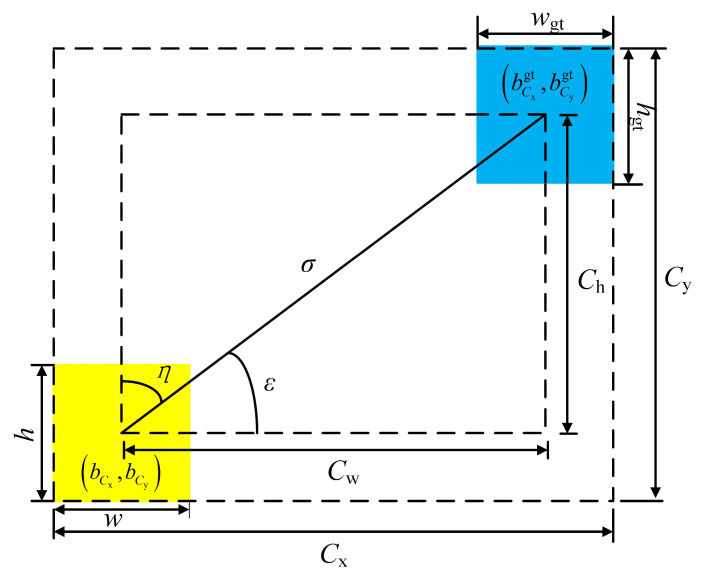
Schematic representation of each parameter in SIoU loss function.

Therefore, in order to solve the above problems, this paper uses the SIoU loss function to replace the CIoU loss function in the original model and redefines the penalty indicator by taking the angle cost into account. Shape loss, IoU loss, angle loss, and distance loss are the four components that make up the SIoU loss function [29]. A schematic representation of the relationship between the parameters in the SIoU loss function is shown in Figure 6, where the yellow box is the true box and the blue box is the predicted box.

The angle loss formula is shown in Equation (1):

(1) $$\Lambda =1-2\times \sin^{2}(\arcsin(x)-\frac{\pi }{4})$$

where

(2) $$x=\frac{C_{h}}{\sigma }=\sin(\varepsilon )$$

(3) $$\sigma =\sqrt{{\left(b_{C_{x}}^{gt}-b_{C_{x}}\right)}^{2}+{\left(b_{C_{y}}^{gt}-b_{C_{y}}\right)}^{2}}$$

(4) $$C_{h}=\max(b_{C_{y}}^{gt},b_{C_{y}})-\min(b_{C_{y}}^{gt},b_{C_{y}})$$

Whether *η* or *ε* is minimized is decided by determining if the angle exceeds 45°. Adding this angle-sensing component minimizes the number of variables associated with distance.

The distance loss formula is shown in Equation (5):

(5) $$\Delta =\sum_{t=x,y}\left(1-e^{-\gamma \rho_{t}}\right)$$

where

(6) $$\rho_{x}={\left(\frac{b_{C_{x}}^{gt}-b_{C_{x}}}{C_{w}}\right)}^{2}$$

(7) $$\rho_{y}={\left(\frac{b_{C_{y}}^{gt}-b_{C_{y}}}{C_{h}}\right)}^{2}$$

(8) γ=2−Λ

In the newly defined penalty indicators, it can be seen that as *ε* approaches 0, the proportion of distance loss in the total loss decreases; on the contrary, as *ε* approaches π/4, the proportion of distance loss increases. In the formula, *ρ*_x_ and *ρ*_y_ are the square of the difference between the width and height, respectively; *γ* is the adjustment factor of angle loss; and, with the increase in the angle, *γ* is given a time-preferred distance value.

The shape loss formula is shown in Equation (9):

(9) $$\Omega =\sum_{t=w,h}{\left(1-e^{-\omega_{t}}\right)}^{\theta }$$

where

(10) $$\omega_{w}=\frac{\left|w-w_{gt}\right|}{\max\left(w,w_{gt}\right)}$$

(11) $$\omega_{h}=\frac{\left|h-h_{gt}\right|}{\max\left(h,h_{gt}\right)}$$

where *θ* is a constant, which can be set at one’s own discretion, and it defines the level of concern for shape loss by optimizing the aspect ratio of the shape, thus limiting the free movement of the shape.

The IoU loss formula is shown in Equation (12):

(12) $$\mathrm{IoU}=\left|\frac{b\cap b^{gt}}{b\cup b^{gt}}\right|$$

In summary, the SIoU loss function formula is shown in Equation (13):

(13) $$L_{\mathrm{SIoU}}=1-\mathrm{IoU}+\frac{\Delta +\Omega }{2}$$

The replaced SIoU loss function solves the problem that the CIoU loss function has the problem that the prediction frames are randomly matched during regression, improves the robustness and generalization ability of the model, and accelerates the convergence speed of the network. However, we found in the actual training process that there was often an imbalance between the hard and easy samples in the dataset, which led to the easy-to-detect samples producing a larger loss value, thus competing with the more hard-to-detect samples. If we did not pay attention to this behavior, the performance of the model after detection would be seriously affected.

**Figure 7:**
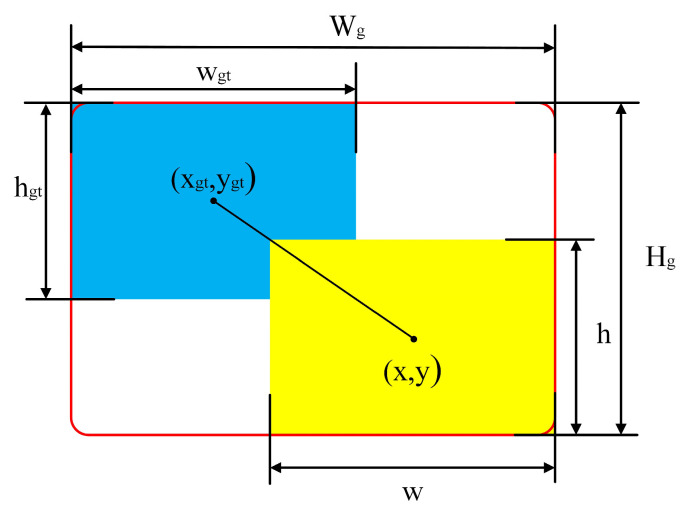
Schematic diagram of each parameter in Wise-IoU loss function.

Therefore, in order to solve the above problems, this paper also introduces the idea of the Wise-IoU loss function on the basis of the SIoU loss function. The Wise-IoU loss function evaluates the quality of anchor boxes by exploiting “outliers” using a dynamic non-monotonic focusing mechanism [30]. When the outlier is small, meaning that the anchor frame is of a high quality, the dynamic non-monotonic focusing mechanism is assigned a smaller gradient gain, while larger outliers are also assigned smaller gradient gains. This strategy not only reduces the competitiveness of high-quality anchor boxes but also reduces the harmful gradients introduced by low-quality samples, making the algorithm more focused on average-quality samples and improving the overall detection performance of the model. The schematic diagram of each parameter in the Wise-IoU loss function is shown in Figure 7. In the figure, the yellow box is the real box, and the blue box is the prediction box.

The formula of the Wise-IoU loss function is shown in Equation (14):

(14) $$L_{\mathrm{WIoU}}=rR_{\mathrm{WIoU}}L_{IoU}$$

where

(15) $$R_{WIoU}=exp\left(\frac{{\left(x-x_{gt}\right)}^{2}+{\left(y-y_{gt}\right)}^{2}}{{\left(W_{g}^{2}+H_{g}^{2}\right)}^{*}}\right)$$

(16) $$r=\frac{\beta }{\delta \alpha^{\beta -\delta }}$$

where R_WIoU_ is the distance focusing mechanism, which is used to enlarge L_IoU_ in the ordinary moderate anchor frame; *r* is the coefficient of the dynamic non-monotonic focusing mechanism, which is used to focus the anchor frame of an ordinary mass; *β* is the outlier used to describe the quality level of the anchor frame; and *α* and *δ* are hyperparameters through which the effects of low- and high-quality anchor frames can be flexibly controlled.

In summary, by the combination of the SIoU loss function and the Wise-IoU loss function ideas, the IoU calculation part in Wise-IoU is replaced by SIoU to obtain the improved Wise-SIoU loss function in this paper, shown in Equation (17):

(17) $$\begin{array}{l} L_{W\mathrm{ise}-SIoU}=rR_{WIoU}L_{SIoU}\\ =r\times exp\left(\frac{{\left(x-x_{gt}\right)}^{2}+{\left(y-y_{gt}\right)}^{2}}{{\left(W_{g}^{2}+H_{g}^{2}\right)}^{*}}\right) \end{array}$$

## 3. Image Acquisition and Preprocessing of Coal Gangue

### 3.1. Image Acquisition Platform for Coal Gangue

**Figure 8:**
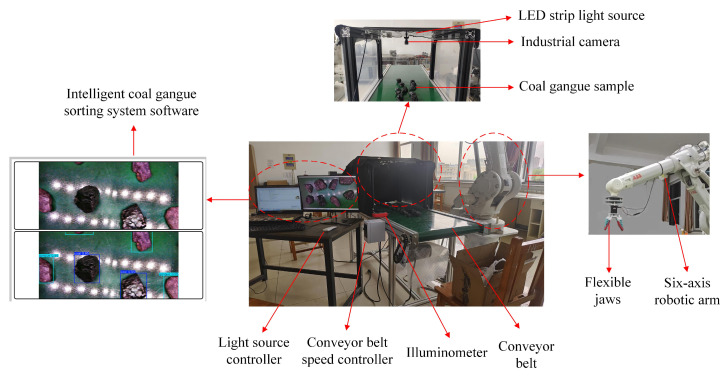
Coal gangue image acquisition platform.

In this paper, considering the influence of low illumination, high noise, and motion blur in the actual coal gangue sorting, we built an experimental platform for coal gangue image acquisition, as seen in Figure 8 below.

The experimental platform mainly included a computer, conveyor belt, conveyor belt speed control device, industrial camera, LED light source, light source controller, and illuminance meter. The camera used an industrial camera with an image sensor of the CMOS type Raspberry Pi UVC (Raspberry Pi Foundation, Cambridge, UK), whose resolution could be manually adjusted according to demand. The light source was a white LED with adjustable brightness. The illuminometer was a Yulide UT383S (UNI-T, Dongguan, China) split digital illuminometer to monitor illuminance, and the detection results could be saved in real-time. The upper computer deployed the intelligent gangue sorting system software developed based on the EMBS-YOLOv8s model, which could detect the gangue in real time and output the detection results.

### 3.2. Image Acquisition of Coal Gangue

**Figure 9:**
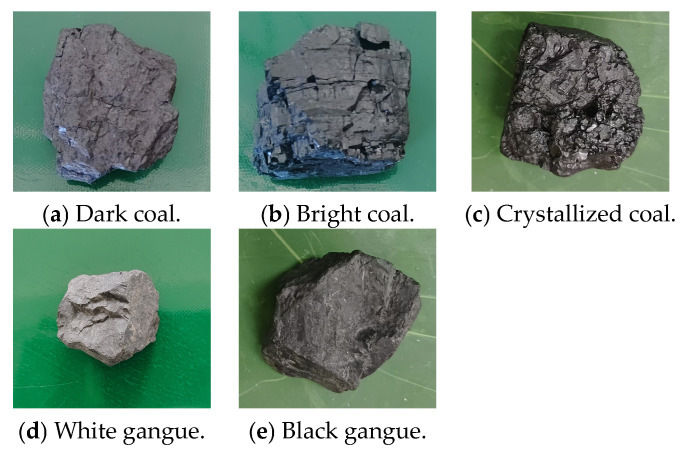
Types of coal and gangue in this experiment.

The coal gangue samples used in this experiment were mainly from the Ordos mining area, and more than 300 samples of coal and gangue were obtained. As shown in Figure 9 below, the coal and gangue were mainly classified into dark coal, bright coal, crystallized coal, white gangue, and black gangue.

**Figure 10:**
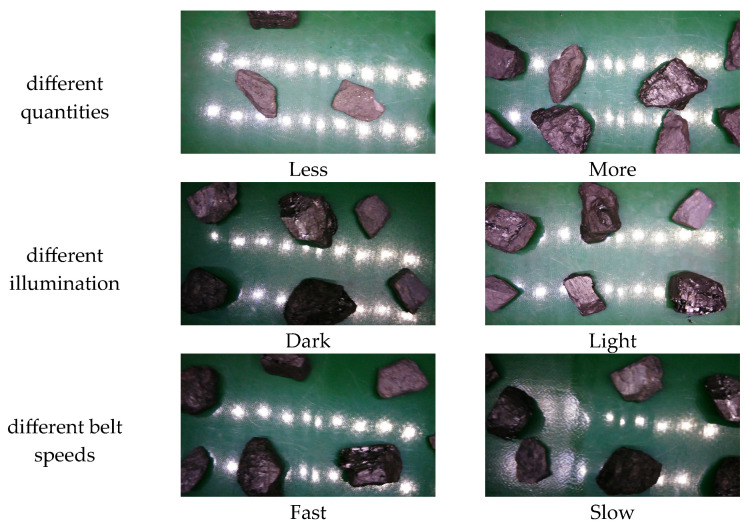
Images of coal gangue collected under different influencing factors.

In order to better highlight the robustness and generalization ability of the improved model proposed in this paper, this paper also simulated the different influencing factors in the actual working environment and collected the coal gangue images under different quantities, different illumination, and different belt speeds, as shown in Figure 10 below. Finally, a total of 2000 coal gangue images were acquired.

### 3.3. Image Preprocessing for Coal Gangue

In the actual working conditions of coal gangue detection, the conveyor belt is often in high-speed operation, and the coal gangue is also affected by illumination, coal dust, and other issues, which leads to the acquisition of fuzzy coal gangue images, parts of regions being brighter or darker, and other issues. Therefore, this paper utilized the CLAHE method to preprocess the coal gangue images [31]. This method could enhance the contrast and clarity of the coal gangue images, improve the brighter or darker regions, and make the outline of coal gangue on the conveyor belt clearer, which was conducive to the training of the model.

**Figure 11:**
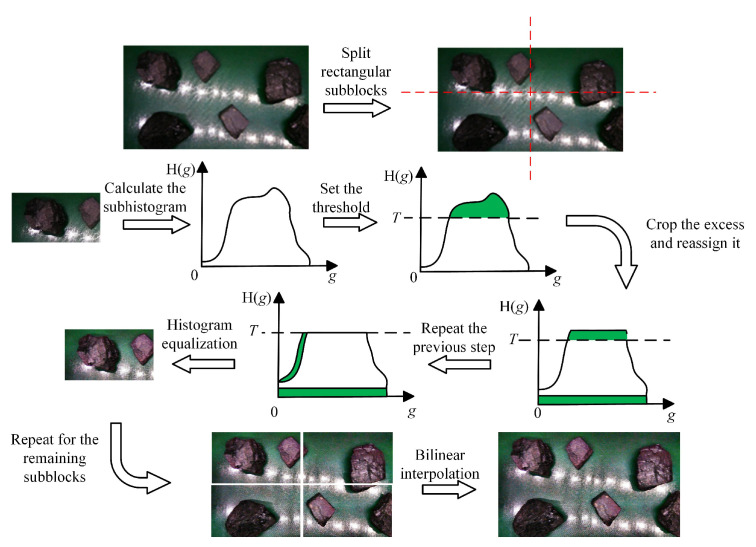
Schematic diagram of CLAHE algorithm.

CLAHE is different from ordinary AHE in that CLAHE limits contrast, thus overcoming the problem of AHE overamplifying noise in the same area of an image. A schematic diagram for the CLAHE algorithm is shown in Figure 11. The CLAHE algorithm flows as follows:

1. Divide the input image into equal and non-overlapping subblocks (the size is usually in pixels).
2. Calculate the histogram H(*g*) of each subblock separately. (*g* indicates the gray level of the current subblock area.)
3. Set a threshold, *T*, for the histogram calculated by each sub-block. A histogram’s gray levels that surpass the threshold are clipped, and the portion that does so is then divided equally among all the histogram’s gray levels. The formula for calculating the threshold *T* is

(18) $$T=T_{\mathrm{clip}}\frac{L_{x}V_{y}}{g}$$

where *L*_x_ and *V*_y_ are the number of pixels in the horizontal direction and vertical direction, respectively; *T*_clip_ is the clipping factor.

The tailoring allocation rules are as follows:

(19) $$S=\sum_{g=0}^{G}(H(x)-T)$$

(20) $$S\_avg=\frac{S}{G}$$

(21) $$H(g)\prime =\left\{\begin{array}{c} H(g)+S\_avg,\;H(g)>T\\ H(g),\;H(g)⩽T \end{array}\right\}$$

where *S* is the total amount of exceededances; *S*_avg is the average of the total number of exceedences; *G* is the possible gray level; and H(*g*)′ is the histogram after redistribution.

4. Perform histogram equalization on the trimmed sub-blocks.
5. The gray value of pixels is reconstructed by a bilinear interpolation algorithm.

**Figure 12:**
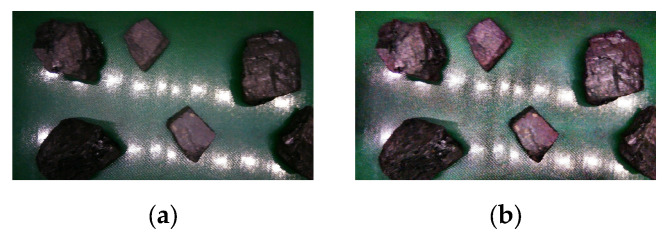
Comparison of coal gangue images before and after CLAHE processing. (**a**) Original image of coal gangue. (**b**) Image of coal gangue after CLAHE processing.

A comparison of the coal gangue images before and after CLAHE processing is shown in Figure 12.

Figure 12a shows the original coal gangue image, which is dark and low-clarity due to low illumination, motion blur, and high noise. Figure 12b shows the coal gangue image enhanced by CLAHE. The clarity and contrast of the coal gangue image enhanced by CLAHE are obviously better than those in the original image.

The value determined by the Laplacian gradient approach was used as the clarity evaluation indicators for the purpose of further assessing the enhancement effect of the CLAHE enhancement algorithm. The image’s clarity increased with the value that was obtained. Additionally, for the purposes of evaluating the image contrast and enhancing the brightness effect, this paper computed the image’s mean and variance in grayscale [32]. The results of the calculations are shown in Table 1 below.

The clarity and contrast of coal gangue images following CLAHE processing increased, as shown in Table 1. A total of 2000 mixed images of coal and gangue were acquired after CLAHE processing.

This paper also took into account that deep learning is more dependent on the number of datasets; the more datasets there are, the higher the network model’s generalization ability is. Therefore, this paper adopted data enhancement methods such as image rotation, flipping, brightness change, noise addition, and blurring to expand the coal gangue dataset. A total of 3200 gangue images were obtained after expansion.

Finally, the coal gangue image dataset was labeled using LabelImg, which set the labels to coal and gangue. This paper labeled using the automatic label method because there were too many labels. Lastly, a 7:1:2 ratio was used to separate the marked dataset into training, verification, and test sets.

## 4. Experimental Environment Configuration and Evaluation Indicators

### 4.1. Experimental Environment Configuration

The software and hardware platform configuration required for model training is shown in Table 2 below.

The uniform input image size for training was 640 × 640, and the batch size was 16. The number of training iteration rounds was 200. The model momentum coefficient was 0.937, the weight decay coefficient was 0.0005, and the initial learning rate was taken as 0.01.

### 4.2. Evaluation Indicators

In this paper, the F1 score and mean average precision (mAP@0.5) are used to measure the detection accuracy effect of the improved model. The number of parameters (Params), floating point operations per second (FLOPs), and model volume (Size)are used to measure the comprehensive performance of the improved model. Frames per second (FPS) are used to evaluate the model’s real-time performance. FPS are the number of image or video frames processed in real time per second. The more FPS there are, the greater the number of images that can be detected in 1s and the better the real-time performance of the model are. The FPS calculation formula is shown in Equation (22) below. The F1 score refers to the reconciled average of precision and recall. The F1 score is calculated as shown in Equation (25) below:

(22) $$FPS=\frac{1000}{\mathrm{preprocess}+\mathrm{inference}+\mathrm{postprocess}}$$

(23) $$P=\frac{TP}{TP+FP}$$

(24) $$R=\frac{TP}{TP+FN}$$

(25) $$F1=\frac{2PR}{P+R}$$

where TP means that the real result is coal and the test result is also coal; TN means that the real result is gangue and the test result is also gangue; FP means that the real result is coal and the test result is gangue; and FN means that the real result is gangue and the test result is coal.

mAP@0.5 is the mean of the average precision (AP) for all classes with an IOU of 0.5. The mAP@0.5 calculation formula is shown in Equation (27) below:

(26) $$AP=\int_{0}^{1}P(R)d(R)$$

(27) $$mAP=\frac{\sum_{i=1}^{K}AP_{i}}{K}$$

where *K* is the number of categories. In the following, mAP@0.5 is represented as mAP for ease of writing.

## 5. Experimental Results and Analysis

### 5.1. Experimental Results of Different Neck Networks

In order to verify the performance of the EMBSFPN structure, this paper analyzed the effect of each structure on the model accuracy and the number of parameters by comparing different neck network structures. The experimental results are shown in Table 3.

As can be seen from Table 3, both structures, the MAFPN [33] and BiFPN, showed an increase in the mAP after replacing the original structure, but the degree of increase was inferior to that of the EMBSFPN structure proposed in this paper. In addition, the reduction in the number of parameters after the replacement of the above two structures was also inferior to that of the EMBSFPN structure. It can be seen that the EMBSFPN structure proposed in this paper could improve the detection accuracy of the model while effectively reducing the number of parameters.

At the same time, in order to show the difference in adding different neck networks more intuitively, the advantages of the proposed structure were reflected. In this paper, Grad-CAM was used to obtain heat maps of the model after passing it through different neck networks [34]. As shown in Figure 11, the darker red color in the graph indicates that the model paid more attention to this part of the image, followed by the yellow color, and the blue color indicates that the model paid the least attention to this part of the image.

**Figure 13:**
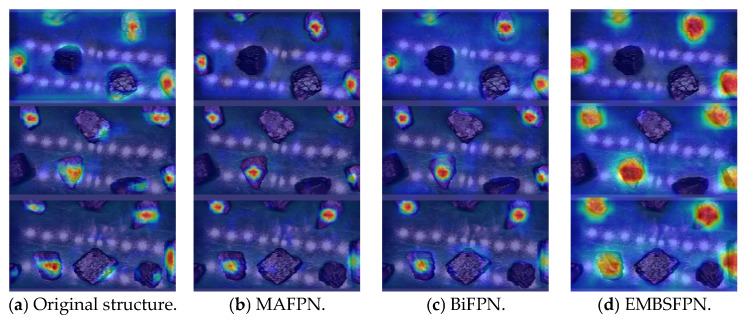
Effect of heat map detection after adding different neck networks.

As can be seen from Figure 13, the original PAN-FPN structure did not fully utilize the features between different scales due to its structural characteristics, and it was difficult to extract effective features for images with complex environments, which led to more cases of misdetection. After replacing the MAFPN structure, BiFPN structure, and EMBSFPN structure, the model paid more attention to the feature extraction of the gangue area, which reduced the false detection rate. However, the comparison found that after replacing the EMBSFPN structure proposed in this paper, the model paid the highest attention to the gangue region, which proves the effectiveness of the EMBSFPN structure proposed in this paper.

### 5.2. Experimental Results of Different Loss Functions

In order to verify the performance of the Wise-SIoU loss function, this paper analyzed the impact of each loss function on the model accuracy and convergence speed by comparing different IoU loss functions. The experimental results are shown in Table 4.

As can be seen in Table 3, the three loss functions of EIoU [35], SIoU, and Wise-IoU had decreased mAP and F1 values compared to the original CIoU loss function. The improved Wise-SIoU loss function, on the other hand, improved, with the highest mAP and F1 values of 96.0% and 90.25%, respectively.

**Figure 14:**
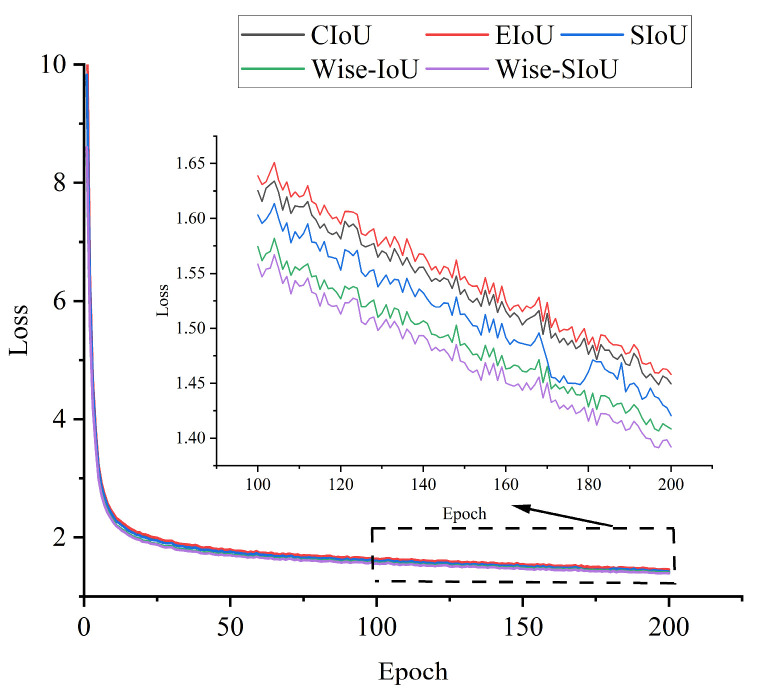
Comparison of loss values of different IOU loss functions on training set.

The total loss curves for different loss functions on the training set are shown in Figure 14. As can be seen in Figure 14, the CIoU loss function used in the original model had a slower decline in loss and possessed the largest total loss value, resulting in a slower convergence of the model. After replacing the CIoU loss function with the SIoU loss function, the total loss value decreased significantly and the rate of loss decrease increased. After finally replacing the Wise-SIoU loss function proposed in this paper, the model achieved the smallest total loss value and the fastest convergence speed. In summary, the Wise-SIoU loss function proposed in this paper maintained high accuracy while also having a faster convergence speed.

### 5.3. Ablation Experiment

In order to better verify the effect of different improvement modules on the performance of the YOLOv8s model, we conducted ablation experiments. The results of the ablation experiments are shown in Table 5.

As can be seen in Table 5, Grouping 1 is the result of the original YOLOv8s model experiment, with an mAP of 93.9%. Grouping 2 is from after the CLAHE processing of the dataset, which improved the contrast and clarity of the images, resulting in a 1.0% increase in the mAP. Grouping 3 is based on Grouping 2, where the original structure was replaced with the EMBSFPN structure so that the feature layers were given different weights to be fused and the features between different scales were fully utilized. The final mAP was improved by 0.9%, and the number of parameters, computation, and volume of the model were all reduced. Despite the relative decline in FPS, the detection speed also remained above 90 f.s^−1^, which was sufficient for real-time detection. Grouping 4 shows the improved EMBS-YOLOv8s model proposed in this paper. The mAP, the number of parameters, the computation, and the volume of this model were all optimized. Compared with the original YOLOv8s model, the mAP was improved by 2.3%, and the number of parameters, computation, and model volume were reduced by 29.59%, 12.7%, and 28.4%, respectively.

**Figure 15:**
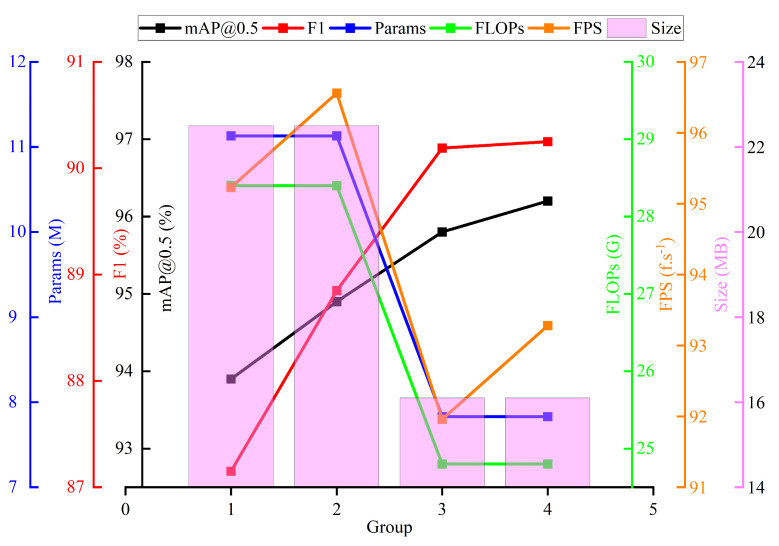
Comparison of ablation experiment results.

Figure 15 shows the specific performance of the model in the mAP, F1, Params, FLOPs, FPS, and model volume after each module was added. As can be seen from the figure, Group 4, that is, the final improved EMBS-YOLOv8s model, additionally increased the model’s detection accuracy while also drastically lowering the number of parameters, computation, and volume of the model. In addition, the model had a detection speed of 93.28 f.s^−1^, which provided some real-time detection performance.

### 5.4. Comparative Experiment

**Figure 16:**
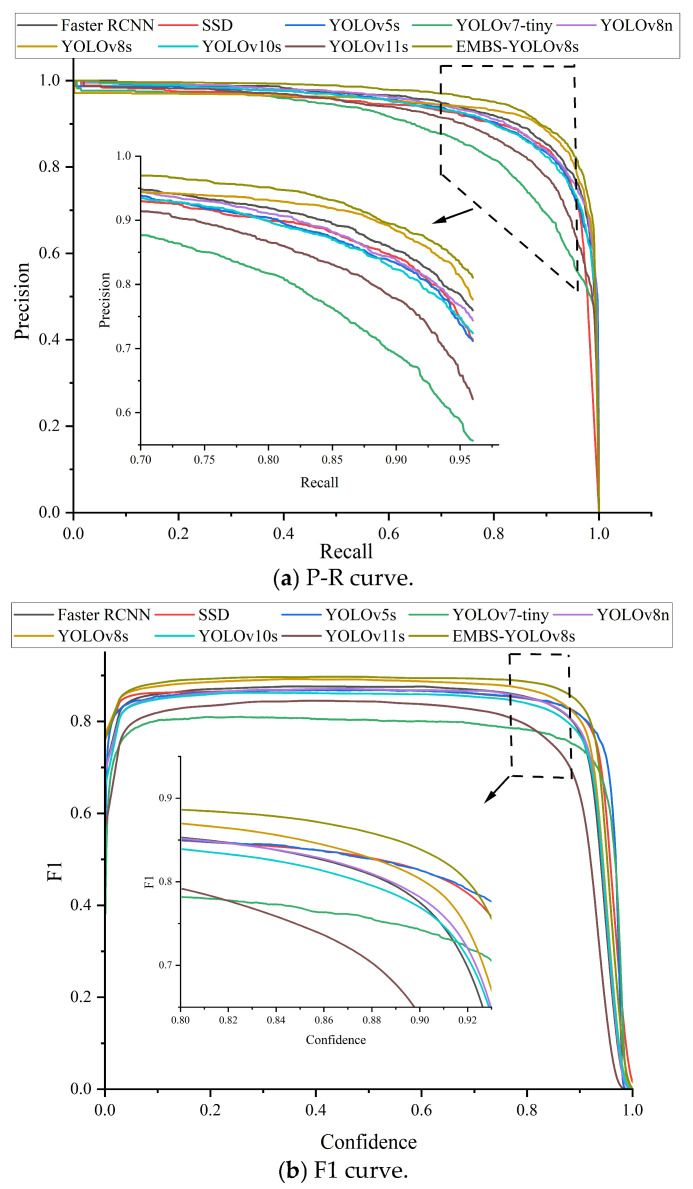
The P-R curve and F1 curve of each model using the test set.

In this paper, we also compared the EMBS-YOLOv8s model with the Faster-RCNN model, the SSD model, the YOLOv5s model, the YOLOv7-tiny model, the YOLOv8n model, the YOLOv8s model, the YOLOv10s model, and the YOLOv11s model in comparison experiments. A comparison of the P-R curves and F1 curves for each model using the test set is shown in Figure 16.

The larger the area enclosed by the P-R curve and the coordinate axes is, the higher the AP value and the higher the mAP value are. From Figure 16a, it can be seen that the improved EMBS-YOLOv8s model has the largest area enclosed by the P-R curve and the coordinate axes compared with other models, which corresponds to a higher AP value and a higher mAP value, so the accuracy is optimal. From Figure 16b, it can be seen that the improved EMBS-YOLOv8s model has the largest area enclosed by the F1 curve and the coordinate axes compared with other models. It indicates that the model in this paper performed better than other models in terms of accuracy and recall in the test set.

Table 6 shows the specific values of the EMBS-YOLOv8s model from the comparison experiments with the Faster-RCNN model, SSD model, YOLOv5s model, YOLOv7-tiny model, YOLOv8n model, YOLOv8s model, YOLOv10s model, and YOLOv11s model. It is visible from the table that the mAP of the EMBS-YOLOv8s model proposed in this paper reached 96.0%, which was 1.8%, 4.1%, 3.0%, 7.5%, 2.5%, 2.1%, 3.3%, and 4.8% higher than that of the Faster-RCNN model, SSD model, YOLOv5s model, YOLOv7-tiny model, YOLOv8n model, YOLOv8s model, YOLOv10s model, and YOLOv11s model, respectively. The F1 score was 90.25%, which was 2.7%, 10.87%, 3.93%, 8.19%, 3.41%, 3.1%, 3.75%, and 5.12% higher than that of the other models, respectively. The number of parameters, FLOPs, and model volume were at a better level compared to in other models, which were 7834599M, 24.8G, and 16.1MB, respectively and reduced by 29.59%, 12.68%, and 28.44% relative to the original YOLOv8s model, respectively. This demonstrates the effectiveness of the improvements proposed in this paper in reducing the number of parameters, model computation, and model volume. Although the FPS measurement was reduced relative to that of the original YOLOv8s model, it was at a better level compared with that in other models, and the current detection speed of 93.28 f.s^−1^ could already meet the requirements of real-time detection.

**Figure 17:**
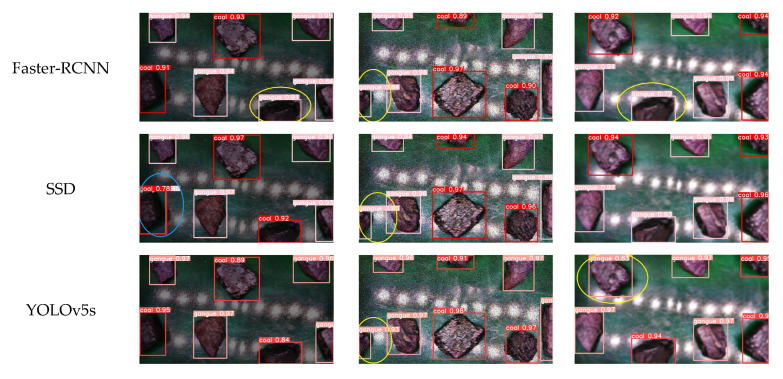

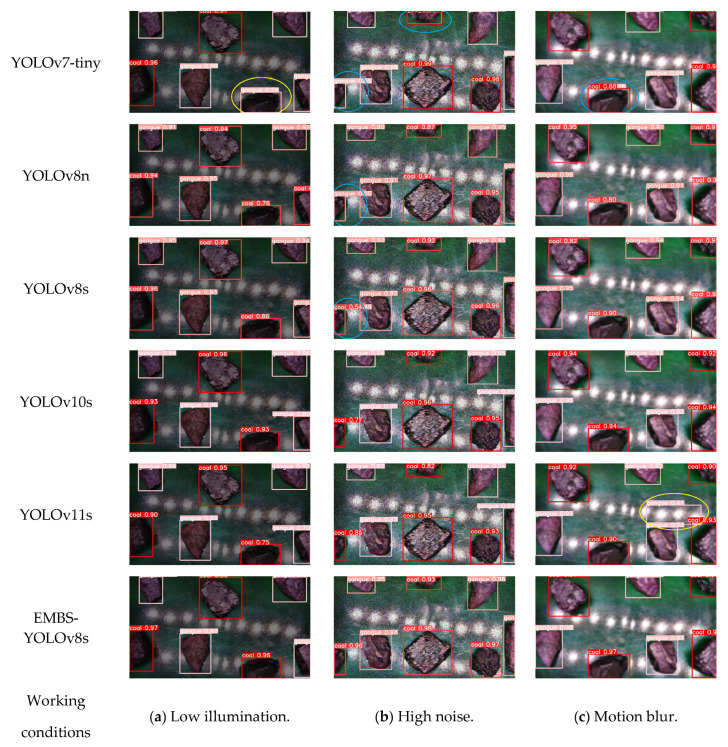
Detection results of different models under the influence of three external factors.

In order to more intuitively reflect the detection effect of the improved EMBS-YOLOv8s model, this paper also compared the detection effect of different models under the influence of three external factors. The results are shown in Figure 17, where the red boxes are marked as coal, the pink boxes are marked as gangue, the yellow ellipses are marked as misdetection, and the blue ellipses are marked as inaccurate predictions.

It can be seen from Figure 17a that in a low-illumination environment, the Faster-RCNN model and the YOLOv7-tiny model had the phenomenon of false detection, the SSD model had the phenomenon of inaccurate prediction, and the other models showed good detection results. However, regarding the detection confidence score, the improved EMBS-YOLOv8s model proposed in this paper had the best confidence score. It can be seen from Figure 17b that in a high-noise environment, the Faster-RCNN model, SSD model and YOLOv5s model had the phenomenon of false detection, and the YOLOv7-tiny, YOLOv8n, and YOLOv8s models had the phenomenon of inaccurate prediction, while the EMBS-YOLOv8s model did not have the above phenomena. This proves that the improved EMBS-YOLOv8s proposed in this paper could effectively improve the detection accuracy of the model. It can be seen from Figure 17c that in the motion-blur environment, the Faster-RCNN model, YOLOv5s model, and YOLOv11s model had the phenomenon of false detection, the YOLOv7-tiny model had the phenomenon of inaccurate prediction, and the other models had normal detection. However, regarding the detection confidence score, the improved EMBS-YOLOv8s model proposed in this paper had the best confidence score. In all the above detection tasks under the influence of complex external factors, the EMBS-YOLOv8s model did not suffer from misdetection, omission, and inaccurate prediction, and the confidence score was high overall, confirming the superiority of the EMBS-YOLOv8s model proposed in this paper.

## 6. Conclusions

(1) A coal gangue detection method based on the EMBS-YOLOv8s model is proposed in this paper. By preprocessing the image with CLAHE, the coal gangue image’s clarity and contrast are enhanced. The EMBSFPN structure replaces the original PAN-FPN structure, increasing detection accuracy while decreasing model complexity. The CIoU loss function is improved with the Wise-SIoU loss function; this fixes the imbalance of hard- and easy-to-detect samples in the dataset in addition to enhancing the model’s convergence and stability.
(2) The experimental results show that the average detection accuracy of the EMBS-YOLOv8s model on the self-constructed coal gangue dataset reached 96.0%, which was 2.1% higher than that of the original YOLOv8s model; the number of parameters, computation, and volume of the model were also reduced by 29.59%, 12.68%, and 28.44%, respectively, relative to those of the original YOLOv8s model. Meanwhile, compared with other YOLO series models, the EMBS-YOLOv8s model had high accuracy, low complexity, and better detection speed, and, at the same time, it could also effectively avoid the occurrence of misdetection and omission in complex scenes such as those with low illumination, low noise, and motion blur.
(3) In future work, we will continue to aim at the coexistence of the lightweight characteristic and high accuracy of the model and study a model that can be deployed in the coal gangue sorting robotic system to verify its performance in the actual intelligent sorting of coal gangue. In addition, if we consider the fusion of visible, infrared, or multispectral images to form a multimodal feature for the model to learn in the future, it will effectively improve the accuracy of the detection of coal gangue in different environments.

## Figures and Tables

**Table 1:** Image clarity and contrast evaluation results.

Evaluation Indicators	Original Image of Coal Gangue	Image of Coal Gangue After CLAHE Processing
Laplacian	1029.69	4103.82
Mean and Variance	1.48—brightness is too dark	0.84—brightness normal

**Table 2:** Software and hardware platform configuration.

Name	Configuration
Operating system	Windows11
Python	3.8.2
Pytorch	2.0.1
CUDA	11.8
CuDNN	11.8

**Table 3:** Comparison of the results of different neck networks.

Neck Network	mAP/%	F1/%	Params/M
Original structure	93.9	87.15	11,126,358
MAFPN	94.6	88.69	11,190,838
BiFPN	94.9	89.71	7,365,090
EMBSFPN	95.8	90.19	7,834,599

**Table 4:** Comparison of results of different IOU loss functions.

IOU Loss Functions	mAP/%	F1/%
CIoU	95.8	90.19
EIoU	94.3	88.05
SIoU	95.0	88.62
Wise-IoU	95.1	89.35
Wise-SIoU	96.0	90.25

**Table 5:** Results of ablation experiments.

Group	1	2	3	4
CLAHE		√	√	√
EMBSFPN			√	√
Wise-SIOU				√
mAP/%	93.9	94.9	95.8	96.0
F1/%	87.15	88.85	90.19	90.25
Params/M	11,126,358	11,126,358	7,834,599	7,834,599
FLOPs/G	28.4	28.4	24.8	24.8
Size/MB	22.5	22.5	16.1	16.1
FPS/f.s^−1^	95.23	96.56	91.96	93.28

“√” indicates that the corresponding module was added.

**Table 6:** Comparative experimental results.

Model	mAP/%	F1/%	Params/M	FLOPs/G	Model Size/MB	FPS/f.s^−1^
Faster-RCNN-Resnet50	92.9	85.55	41,755,286	134.38	108.10	23.21
SSD-Vgg	90.6	79.38	35,641,826	34.86	91.09	80.17
YOLOv5s	93.0	86.32	7,015,519	15.8	14.5	88.91
YOLOv7-tiny	88.5	82.06	6,010,302	13.0	12.3	102.15
YOLOv8n	93.5	86.84	3,006,038	8.4	6.3	98.34
YOLOv8s	93.9	87.15	11,126,358	28.4	22.5	95.23
YOLOv10s	92.7	86.50	8,036,508	24.4	16.6	91.45
YOLOv11s	91.0	85.13	9,413,574	21.3	19.2	86.70
EMBS-YOLOv8s	96.0	90.25	7,834,599	24.8	16.1	93.28

## Data Availability

The experimental data used to support the findings of this study are included within the article.
